# Long-term clinical and bacterial effects of xylitol on patients with fixed orthodontic appliances

**DOI:** 10.1186/s40510-015-0103-z

**Published:** 2015-10-14

**Authors:** Mohamed I. Masoud, Reem Allarakia, Najlaa M. Alamoudi, Romesh Nalliah, Veerasathpurush Allareddy

**Affiliations:** Department of Developmental Biology, Harvard School of Dental Medicine, Boston, MA USA; Orthodontics Department, King Abdulaziz University, Jeddah, Saudi Arabia; Pediatric Dentistry Department, Faculty of Dentistry, King Abdulaziz University, Jeddah, Saudi Arabia; Pediatric Dentistry, Armed Forces Hospital, Jeddah, Saudi Arabia; College of Dentistry, University of Michigan at Ann Arbor, Ann Arbor, MI USA; Department of Orthodontics, College of Dentistry, The University of Iowa, Iowa City, IA 52242 USA

**Keywords:** Xylitol, Fixed orthodontic appliances, Oral hygiene, Plaque, Bacterial counts

## Abstract

**Background:**

The objective of this study was to evaluate long-term clinical and bacterial effects of using 6 g of xylitol per day for 3 months on patients with full fixed orthodontic appliances.

**Methods:**

The study was a pilot clinical trial that included 41 subjects who were undergoing orthodontic treatment. The subjects were randomly divided into three groups. Group A received xylitol chewing gum, group B received xylitol dissolvable chewable tablets, and Group C served as the control group and did not receive xylitol gums or tablets. Clinical examination and the collection of plaque and saliva samples were carried out at baseline and 3, 6, and 12 months. All three groups were given oral hygiene instruction and were put on a 6-month cleaning and topical fluoride schedule. Plaque scores and bacterial counts were used to evaluate the effectiveness of the different approaches at reducing the caries risk.

**Results:**

Xylitol groups did not experience any more reduction in plaque score, plaque MS counts, or salivary MS counts than the control group nor did they have lower values at any of the time points. Chewing gum did not significantly increase the incidence of debonded brackets over the other groups.

**Conclusions:**

Xylitol does not have a clinical or bacterial benefit in patients with fixed orthodontic appliances. Oral hygiene instructions and 6-month topical fluoride application were effective at reducing plaque scores and bacterial counts in patients with full fixed appliances regardless of whether or not xylitol was used.

## Background

Dental caries is a significant public health problem for many developing countries [1, 2]. Studies have shown that orthodontic appliances create retentive areas for plaque and make adequate oral hygiene more difficult to maintain, which subsequently leads to a significantly higher incidence of decalcification [3, 4]. Fixed orthodontic appliances have also been associated with increased concentrations of plaque and saliva Mutans Streptococci (MS) which play a major role in the development of dental caries [5–8].

Preventive strategies traditionally tend to focus on dietary modification and use of fluoride and pit and fissure sealants. Use of antibacterial agents in subjects harboring high levels of MS seems to be a promising modality of caries prevention. Chlorhexidine mouth rinses (CHX) have demonstrated their ability to suppress MS to very low [9, 10]. However, studies of CHX treatment revealed both variability in MS response and incomplete suppression for prolonged periods [11, 12]. Recent caries research has focused on Xylitol, which is a caloric sugar substitute [13, 14]. Most plaque bacteria lack the ability to ferment xylitol into cariogenic end products. Instead, xylitol accumulates intracellularly in MS as a nonmetabolizable metabolite thus inhibiting bacterial growth, reducing their number and reducing the amount of plaque. Xylitol’s presence in the oral environment also selects for less virulent MS populations referred to as xylitol-resistant MS [13–17]. Sorbitol-containing chewing gum has also been shown to reduce the risk of developing caries. However, Sorbitol has no effect on MS and has thus been proven to be less effective than xylitol-containing chewing gum in the prevention of dental caries [16, 18–20].

Milgrom et al. studied the dose response relationship between MS and xylitol chewing gums. The results demonstrated that MS numbers decreased as daily xylitol intake increased with a plateau effect occurring between 6.88 and 10.32 g of xylitol per day [21]. The use of xylitol on orthodontic patients has been studied by several researchers with somewhat conflicting conclusions [22–24]. It is unclear whether the conflicting conclusions are due to variations in xylitol dosage, method of delivery, or follow-up period.

The objectives of this pilot study are as follows:

- To evaluate the effect of different xylitol delivery vehicles providing a dose of 6 g per day on MS counts in plaque and saliva.
- To compare the effect of the different xylitol delivery methods to a control group that was placed on a strict oral hygiene and topical fluoride application program.
- To compare plaque scores, plaque MS counts, and salivary MS counts, in the control and experimental groups.
- To study the long-term effects of xylitol on plaque and saliva MS after xylitol use has been discontinued.
- To evaluate the negative effects of the different vehicles on the orthodontic appliances (loose brackets and bands).

## Methods

### Study design and inclusion criteria

This study is a pilot clinical trial of 41 adolescents and young adults of both sexes that were undergoing orthodontic treatment with fixed appliance between January and December 2009. The patients’ ages ranged between 12 and 30 years (mean = 18.4 years).

The inclusion criteria were as follows:

1. Patients undergoing orthodontic treatment with full fixed appliances
2. Estimated remaining time in orthodontic treatment expected to be greater than 12 months
3. No significant medical history
4. Dental history free from temporomandibular joint (TMJ) disorders
5. No active decay

### Informed consent and institutional review board approval

After being given verbal and written information about the study, all volunteers signed informed consent forms if they were above the age of 18. If the subjects were under 18, one of the parents signed a consent form and the child provided his or her assent. The study protocol and consent forms were approved by the ethical committee at King Abdulaziz University, Jeddah, Saudi Arabia. The study was not registered on clinicaltrials.gov since it falls into one of the categories that is excluded from the registration and results submission requirement under FDAAA 801. Xylitol is not considered as a drug and is categorized as a food additive by the US Food and Drug Administration [25].

### Study protocol

The groups were randomly assigned to one of three groups. A sequence of numbers were generated, and each number was assigned to a treatment group. These numbers were placed in sealed envelopes along with the examination sheets. When a patient agreed to be enrolled in the study, they got a sealed envelope which had the assignment sheets and the group to which they were assigned to. Only the study administrator (who was not an orthodontist) was aware of the unique identifier (a number written on the cover of the envelope) that linked each patient to the treatment group. Patients were not blinded since there was no group that got a sugar pill, and they could tell whether they were receiving mints or gum. The orthodontist was not involved in the research process and would not know which group the patient was in.

**Fig. 1:**
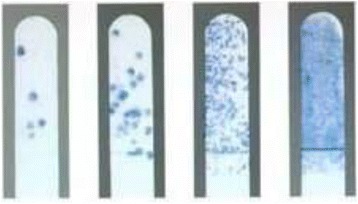
Dentocult SM bacterial count model chart

Patients in group A (*n* = 13) were asked to consume 6 pieces of xylitol chewing gum per day for 3 months (2 pieces 3 times a day after breakfast, lunch, and dinner). The patients were asked to chew the gum for no less than 5 min. Every piece contained 1 g of xylitol resulting in a daily dose of 6 g of xylitol. Patients in group B (*n* = 13) were asked to consume 12 pieces of xylitol chewable mints per day for 3 months (4 pieces 3 times a day after breakfast, lunch, and dinner). Every piece of mint contained 0.5 g of xylitol so group B also received 6 g of xylitol per day. Patients in group C (*n* = 12) served as controls with no intake of xylitol gum or mints. Compliance was evaluated by asking the patients if they adhered to the prescribed doses and by asking them to bring empty containers. If they were consistently not able to produce them or reported not using the xylitol, they were omitted from the study. Clinical examination was carried out at base line, 3 months, 6 months, and 12 months to evaluate TMJ, caries, labial decalcification, number of broken brackets, plaque scores, and gingival scores. For TMJ, the parameters evaluated included the following: muscle of mastication (temporalis and masseter) sensitivity to palpitation, TMJ pain upon palpation during opening and/or closing, maximum opening, and joint noise [26]. Caries evaluation was done using a probe, a dental mirror, and a dental chair light. The recording system was based on the WHO criteria from 1987 [27]. The codes used were as follows: healthy surfaces as well as initial carious lesions without clinically detectable loss of substance were coded as 0, enamel caries with loss of substance as 1, filled surfaces as 2, missing due to caries as 3, and any surfaces with sealant or crown and bridge or not present or extracted for any reason except caries were excluded as 4. Labial decalcification was evaluated by drying the teeth and counting areas with visible white areas [28]. The number of broken brackets was assessed by having the treating orthodontist report the number of broken brackets at the beginning of each visit. Plaque scores were determined using the plaque component of the Simplified oral debris index (DI-S) by Greene and Vermillion [29]. Six tooth surfaces were examined: the buccal surface of the upper right first molar (16 B), the buccal surface of upper right central incisor (11B), the buccal surface of upper left first molar (26B), the lingual surface of right lower first molar (46B), the buccal surface of lower left central incisor (31B), and the lingual surface of left first lower molar (36L). Each surface was scored according to the amount of the plaque covering the tooth surface. If there was no plaque covering the tooth surface, it was scored as 0. If plaque covered less than 1/3 of the crown, it was scored as 1. If plaque covered more than 1/3 of the crown but less than 2/3 of the crown, it was scored as 2. Finally, if plaque covered more than 2/3 of the crown, it was scored as 3. These scores were then added and divided by 6. The Gingival score was determined using the criteria for the gingival index by Loe [30], which is based on gingival condition, color, and bleeding on probing. For normal gingival, 0 was scored. A score of 1 was used to indicate mild gingival inflammation, a slight change in color, slight edema but no bleeding on probing. A score of 2 was used to indicate gingival inflammation with moderate color change, edema, and bleeding on probing. A score of 3 indicated severe inflammation, marked redness, edema, and bleeding upon probing. Plaque and saliva mutans streptococci (MS) levels were measured using Dentocult SMTM (Orion Diagnostica, Helsinki, Finland). The method is based on the use of a selective culture broth and the adherence and growth of mutans streptococcus bacteria on the test strip. The Dentocult MS strip mutans test was performed at the beginning of enrollment, at 3-month follow-up, at 6-month follow-up, and 12 months after enrollment. Patients were told to avoid eating, smoking, toothbrushing, and using mouth wash 2 h before the collection of the plaque and saliva samples. A bacitracin disc was placed in the selective culture broth for 15 min before sampling. Plaque samples were collected using a dental probe and were spread thoroughly but gently on the rough surface on the strips. Four sites were simultaneously sampled (the buccal surface of the upper right central incisor, the buccal surface of the upper left first molar, the buccal surface of the lower left central incisor, and the lingual surface of the lower right first molar). The patients were asked to chew a paraffin pellet for 1 min to stimulate the secretion of saliva and transfer the bacteria from the tooth surfaces to the saliva. The patients were instructed to swallow any excess saliva then pass the rough surface of the strip on the tongue to collect the remaining saliva. The strips were then placed in the selective culture broth and incubated in an upright position at 37 °C for 48 h with the cap one quarter of a turn open. The quantification of mutans streptococci was done after the incubation, and the presence of mutans streptococci (MS) was detected by observing dark blue to light blue raised colonies on the inoculated strips. Mutans streptococci colonies were differentiated from the colored plaque debris by their elevated surfaces. In saliva sampling, the mutans streptococci adhered to the rough surface of the strip in proportion to their density in saliva. The density was compared with the model chart provided by the kit manufacturer and categorized into four classes (Fig. 1): The first category in the chart was “Class 0” which indicates less than 10,000 colony-forming units (CFU) per milliliter. Class 1 indicates less than 100,000 CFU/ml. Class 2 indicates between 100,000 and 1000,000 CFU/ml, and Class 3 indicates greater than 1,000,000 CFU/ml.

### Statistical analysis

The data was subjected to analysis of variance for repeated measures. The intraexaminer reliability was carried out according to the Kappa reliability tests, on five randomly selected patients at baseline and 3, 6, and 12 months. There was almost perfect agreement across the five patients between the 1st and 2nd examination results with an average kappa score of =0.984. The baseline and the post-intervention MS counts in plaque and saliva as well as the mean plaque scores and other variables like the number of debonded brackets, DMFT were measured in the control group and test groups. Proportions were compared using Wilcoxon’s signed rank test, and *P* < 0.05 was used to indicate the statistical significance. The test used for group comparisons was the Kruskal-Wallis test using the same level of significance. All statistical analyses were performed using SPSS version 22.0 software (IBM Corp, New York).

## Results

The results from this pilot study are presented in Tables 1, 2, 3, 4, and 5. The sample included 41 participants at the baseline, 36 participants at 3-month follow-up, 31 participants at 6 months, and 31 participants 12 months. The 3-month attrition of the sample was due to poor compliance with using the xylitol, and the 6- and 12-month attrition was due to unanticipated discontinuation of orthodontic treatment.

**Table 1:** Demographic description of the study sample at base line

	Age years (SD)	Gender	
		M *n* (%)	F *n* (%)
Gum	17.79	3	11
*n* = 14	(±4.594)	−21.4	−78.6
Mint		4	10
	16.86 (±3.483)		
*n* = 14		−28.6	−71.4
Control		2	11
	18.77 (±5.585)		
*n* = 13		−15.4	−84.6
*P* value	0.153	0.709

**Table 2:** Comparison^a^ of outcomes based on surfaces

Variables	From baseline to	Group					
		Gum		Mint		Control	
		Z	*P* value	Z	*P* value	Z	*P* value
Plaque	3rd month	−3.274^b^	0.001	−3.611^b^	0	−3.123^b^	0.002
	6th month	−0.423^b^	0.672	−0.570^b^	0.568	−2.600^b^	0.009
	12th month	−1.890^b^	0.059	−0.756^b^	0.45	−2.646^b^	0.008
Plaque MS counts	3rd month	−1.763^b^	0.078	−2.313^b^	0.021	−2.979^b^	0.003
	6th month	−1.500^b^	0.134	−2.786^b^	0.005	−2.938^b^	0.003
	12th month	−2.082^b^	0.037	−1.877^b^	0.061	−3.002^b^	0.003
Salivary MS counts	3rd month	−0.351^c^	0.725	−1.421^b^	0.155	−1.265^b^	0.206
	6th month	−0.531^b^	0.595	−0.816^c^	0.414	−0.216^c^	0.829
	12th month	−1.342^c^	0.18	−0.879^c^	0.38	0.000^d^	1

^a^Wilcoxon Signed-Rank Test

^b^Based on positive ranks (decreased score)

^c^Based on negative ranks (increased score)

^d^The sum of negative ranks equals the sum of positive ranks (no change)

**Table 3:** Summary of results from Kruskal-Wallis test

Variables	Period	Chi-square	*Df*	*P* value
Plaque	Baseline—Plaque	1.126	2	0.57
	3rd month—plaque	0.945	2	0.624
	6th month—plaque	4.815	2	0.09
	12th month—plaque	8.687	2	0.013
Plaque MS counts	Baseline—MS counts	1.36	2	0.507
	3rd month—MS counts	8.316	2	0.016
	6th month—MS counts	4.851	2	0.088
	12th month—MS counts	4.309	2	0.116
Salivary MS counts	Baseline—saliva	4.432	2	0.109
	3rd month—saliva	2.535	2	0.282
	6th month—saliva	0.968	2	0.616
	12th month—saliva	2.993	2	0.224

**Table 4:** Results from Mann–Whitney Test for Plaque—12 months

Statistics	Gum vs mint	Gum vs control	Mint vs control
Remarks	Mint is higher	No difference	Mint is higher
Z	−2.429	−0.16	−2.508
*P* value	0.015	0.873	0.012

**Table 5:** Results from Mann–Whitney Test for MS counts—3rd month

Statistics	Gum vs mint	Gum vs control	Mint vs control
Remarks	No difference	Gum is higher	Mint is higher
Z	−0.582	−2.119	−2.846
*P* value	0.56	0.034	0.004

The baseline characteristics of the patients are summarized in Table 1. The mean age of those in the gum group was 17.8 years compared to 16.9 years in mint group and 18.8 years in the control group. A total of 9 males and 32 females participated in the study. Overall, there were no statistically significant differences in ages and distribution of gender between the three groups.

Comparison of the plaque, MS counts, and Saliva scores across different time points from baseline are presented in Table 2, and estimates from the Kruskal-Wallis test are presented in Table 3. The first parameter that was evaluated was the mean plaque score: All three groups had a reduction in plaque scores that continued throughout the 12-month period. This was only significant at the 3-month time point for the mint and gum groups. For the control group, the decrease in plaque score compared to the baseline readings was significant at the 3-, 6-, and 12-month follow-up periods. There was a statistically significant difference between the groups’ plaque scores only at the 12-month time point with the gum group and control group having lower plaque scores than the mint group with no statistically significant difference between the gum and control groups (Table 4). None of the other time points has a statistically significant difference in plaque score between the groups.

The second parameter evaluated was the MS count in plaque: All three groups had a reduction in plaque MS counts compared to the baseline readings at all the time points. This was statistically significant for the mint group at 3 and 6 months. It also approached statistical significance at 12 months. Compared to the baseline values, the gum group had a reduction in plaque MS counts that was only significant at the 12-month time point. For the control group, the reduction plaque MS counts compared to baseline values was statistically significant at all three follow-up time points. There was a statistically significant difference between the groups in plaque MS counts at 3 months with the control group having lower values than the other two groups (Table 5).

The third parameter that was evaluated was the MS count in saliva. The salivary MS counts also decreased compared to baseline values in all the groups at all three follow-up time points, but this was not statistically significant. There was also no statistically significant difference in salivary MS count between the groups.

There was no statistically significant difference in the DMFT scores, TMJ evaluation parameters, or broken brackets between the groups or among the groups for any of the time intervals that were evaluated.

## Discussion

The present pilot study was undertaken to evaluate the effectiveness of 6 g/day dose of xylitol on the ecology of dental plaque and saliva in everyday clinical orthodontic practice in patients with fixed orthodontic appliances. Since orthodontists would be expected to use this method in conjunction with standard oral hygiene instructions, routine dental cleanings, and topical fluoride application, we decided to make sure both the test and control groups received such measures. Like most previous studies, our results demonstrated consistent reduction in plaque scores, MS counts in plaque, and MS counts in saliva with the use of xylitol. However, our control group also had a similar reduction with neither xylitol group having significantly lower levels at any of the observed time points. Isotupa et al. had no control group that received no sugar alcohol and mentioned that all of their groups were given the same oral hygiene instructions given to all orthodontic patients [22]. They did not report any additional measures to insure that the patients went to their routine cleaning visits or had topical fluoride application. They reported a significant reduction in plaque and salivary MS counts in the xylitol groups but not the Sorbitol-only group. They did not compare the groups to each other at base line or follow-up. They also only followed patients up to 4 weeks and asked patients to refrain from any oral hygiene practices for 3 days prior to sample collection. Isotupa et al. also used a higher daily dose of xylitol that we used in this study [22]. We chose our dose based on the dose/response study by Milgrom et al. [21]. They concluded that MS counts decreased as daily xylitol dose decreased with a plateau occurring above 6.88 g/day [21]. We also felt that compliance would become more challenging for patients if we asked them to consume more than 6 g/day (6 pieces of gum or 12 pieces of mint). These differences in protocol and statistical tests reported are probably the reason for the different conclusions. Stecksén-Blicks et al. used a maximum dose that was lower than ours (3.4 g/day) [23]. Like our study, their design included a group that did not use any form of sugar alcohol. However, they did not report administering topical fluoride, scheduling regular cleanings, or oral hygiene instructions for any of their groups. Like Isotupa et al., they did not compare the MS levels of the different groups to each other at each time point [22, 23]. They reported no difference in plaque MS counts at 6, 8, and 18 weeks. They did however report a small but statistically significant difference in salivary MS counts at 6 weeks that disappeared at 8 and 18 weeks. Surprisingly, this was observed with the 1.7 g/day group and not the 3.4 g/day group. Our first observation was 12 weeks, so we might have missed the temporary reduction in MS counts that they observed. We believe that the protocol we have chosen is more consistent with what would be relevant in a clinical setting. If good oral hygiene instructions, and regular cleanings and topical fluoride application, can eliminate the measurable effect of xylitol, and if the effect of xylitol is not sustainable during a 3-month period of use, then there does not seem to be a point in prescribing it as a measure for caries prevention.

Our study did not measure salivary buffering capacity or the proportion of xylitol-resistant MS strain, so there may be a benefit to xylitol that our study design did not account for.

For DMFT, labial decalcification, TMJ evaluation, mean plaque score, plaque and salivary MS counts, and broken brackets, there were no statistically significant differences between the groups at any of the time points.

Xylitol has been extensively studied in recent years, and all clinical studies concerning the effects of xylitol on caries development agree on its non carcinogenicity and on the beneficial effect of substituting sucrose with xylitol in chewing gum and sweets [11]. Our results demonstrated that xylitol chewing gum and chewable tablets had no negative effects on orthodontic appliances and did not increase risk of developing cavities. This is consistent with previous studies. These consistent findings across multiple studies question the “no chewing gum” instructions most orthodontic practices provide [21–23]. Despite the non cariogenicity of xylitol, we are unable to advocate its use as a caries prevention measure since it did not provide any measurable benefit over a control group that received oral hygiene instructions, regular cleanings, and topical fluoride application.

Among the drawbacks of our study design is that our subjects were not blinded since the control group obviously knew that they were not receiving the xylitol products described in the informed consent [31]. This could have potentially encouraged them to take home care more seriously than the other two groups and could have affected our results. We also had a limited sample size that experienced some attrition during the 12-month follow-up period.

## Conclusions

Patients with full fixed orthodontic appliances can use xylitol chewing gum and mints during treatment, but we cannot advocate its use as a caries control measure in an orthodontic setting.

The main conclusions from this pilot study are as follows:

1. Xylitol does not have an effect on plaque score, plaque MS counts, or salivary MS counts in patients with full fixed orthodontic appliances.
2. Chewing gum does not cause any increase to the risk of debonded orthodontic appliances.
3. Oral hygiene instructions and 6-month topical fluoride application were effective at reducing plaque scores in patients with full fixed appliances regardless of whether or not xylitol was used.
